# Mobilization of Stored Iron in Mammals: A Review

**DOI:** 10.3390/nu5104022

**Published:** 2013-10-10

**Authors:** Maria C. Linder

**Affiliations:** Department of Chemistry and Biochemistry, California State University, Fullerton, CA 92834-6866, USA; E-Mail: mlinder@fullerton.edu; Tel.: +1-657-278-2472; Fax: +1-657-278-5316

**Keywords:** iron stores, ferritin, iron mobilization, lysosomes, proteasome, autophagy, DMT1, ferroportin, erythrocyte iron recycling, hepcidin, erythropoietin

## Abstract

From the nutritional standpoint, several aspects of the biochemistry and physiology of iron are unique. In stark contrast to most other elements, most of the iron in mammals is in the blood attached to red blood cell hemoglobin and transporting oxygen to cells for oxidative phosphorylation and other purposes. Controlled and uncontrolled blood loss thus has a major impact on iron availability. Also, in contrast to most other nutrients, iron is poorly absorbed and poorly excreted. Moreover, amounts absorbed (~1 mg/day in adults) are much less than the total iron (~20 mg/day) cycling into and out of hemoglobin, involving bone marrow erythropoiesis and reticuloendothelial cell degradation of aged red cells. In the face of uncertainties in iron bioavailability, the mammalian organism has evolved a complex system to retain and store iron not immediately in use, and to make that iron available when and where it is needed. Iron is stored innocuously in the large hollow protein, ferritin, particularly in cells of the liver, spleen and bone marrow. Our current understanding of the molecular, cellular and physiological mechanisms by which this stored iron in ferritin is mobilized and distributed—within the cell or to other organs—is the subject of this review.

## 1. Introduction

Iron is traditionally considered the most abundant trace element in the mammalian organism, the total iron in adult humans being in the range of 2.5–4.5 g. These quantities are much lower than those of the major minerals (Ca, P, K, Na, Cl and Mg), which range from 1200 to 26 g in the human adult [1]. Quantities of iron are only slightly higher than fluorine (~2.6–4 g), and considerably higher than zinc (1.6–2.3 g), silicon (1.1 g), copper (0.11 g) and the many other elements present in the whole organism. Iron is unique in several other ways. For one, it is probably the only element located mainly in the blood (in red blood cell hemoglobin, to mediate oxygen transport), which makes it unusually vulnerable to being lost by bleeding. For another, its chemistry makes it difficult to absorb from the diet, since iron ions form insoluble oxides, hydroxides and other precipitating salts in aqueous solutions at physiological pH; and only nutrients that chelate iron keep some soluble available for uptake by enterocytes. About 10% of dietary non-heme iron is absorbed. Heme iron is absorbed somewhat more efficiently and as the heme complex [2]. Iron stored by plant and animal cells in the large protein ferritin may be absorbed even more efficiently, and by receptor mediated endocytosis [3]. Also unusual is that the amounts of iron absorbed daily (1–2 mg) are only a tiny fraction of the total in the body, and more than an order of magnitude less than the amounts of iron cycling daily in and out of iron-dependent proteins (18–24 mg) [1]. In biological systems, iron is in the 2+ and 3+ state, hemoglobin iron being Fe^2+^, iron transported on transferrin in the blood or stored intracellularly in ferritin being Fe^3+^, and that in enzymes and cytochromes being redox active and cycling between the two states.

Not only is iron difficult to absorb, but the mammal is not well equipped to rid itself of excess iron except by deliberate (or accidental) bleeding. Mechanisms within the organism are poised to ensure that once iron has entered cell fluids and tissues, it is retained. Thus, for example, aged red cells are taken up by macrophages, particularly in spleen and liver, to process the iron within a regulated manner, and recycle it via transporter/transport proteins or store it within the large hollow, multi-subunit protein ferritin. If erythrocytes are lysed within the blood, their hemoglobin binds to haptoglobin in the plasma and is taken to hepatocytes. Any free heme is chaperoned to hepatocytes by hemopexin [4]. Binding to larger proteins ensures that little or no iron is lost by glomerular filtration and entry into urine. Opposing these tendencies to retain iron are natural as well as accidental events that remove body iron. These include menstrual bleeding (4–37 mg per period), pregnancy (350–450 mg total), and accidental bleeding (0.5 mg/mL blood) [1]. Excretion is thought to occur primarily through the sloughing off of enterocytes at the ends of intestinal villi, and a small proportion (about 0.2 mg) is lost in the urine, the origin of which is unclear. More iron is lost when body stores are high, since more is retained by the enterocytes stored in ferritin, in response to signals that the body has sufficient iron. This response is at least partly programmed into enterocytes when they are being formed in crypts at the base of the intestinal villi. New cells migrate to the villus tips in 3–5 days. Shed cells are digested and most of the iron not reabsorbed. Less iron is lost this way when stores are low, since the enterocytes transfer almost all they absorb to the blood and have little or none stored in ferritin.

Apart from iron circulating in adult erythrocyte hemoglobin (2–2.5 g), a significant fraction (~300 mg) is in skeletal and cardiac muscle myoglobin to mediate intracellular oxygen transport [1,5]. Much smaller portions of total body iron are associated with cytochromes in the electron transport system of mitochondria in all cells, and with a limited number of enzymes. The latter include the heme enzymes catalase and cytochrome P450s (“drug metabolizing” enzymes in the endoplasmic reticulum), as well as those requiring iron-sulfur clusters (aconitase) or other complexes. An iron-dependent rate-limiting enzyme for cell division (in all organisms including humans) is ribonucleotide reductase, which provides the deoxyribonucleotides needed for DNA synthesis, and contains a bi-nuclear Fe-O-Fe unit that helps to produce a tyrosyl radical for the reaction. Much smaller quantities enter and exit all other iron dependent proteins. Quantitatively, most of the iron metabolized daily is that being incorporated into developing reticulocytes (in the bone marrow) and removed from aged erythrocytes that have entered reticuloendothelial cells (macrophages that are mostly in spleen and liver). (Macrophages normally account for ~600 mg of body iron [5]). Much of the iron released from cell and protein degradation is at least temporarily stored in ferritin, amounts in ferritin varying enormously from one individual to another [1].

The need for erythropoiesis thus drives much of iron metabolism, and this is regulated especially by the peptide hormone erythropoietin, which responds to oxygen tension in the blood. Thus, if blood is lost, levels of oxygen in blood are reduced and erythropoiesis is stimulated, which requires the mammal to send more iron to the bone marrow. The iron comes from iron stores in ferritin, which tend to be highest in liver, spleen and bone marrow. The iron also comes from enhanced absorption by the intestine, if the dietary iron is chemically available, dissolved in gut fluids. Erythropoietin is produced by the kidney and to some extent also by the liver [6]. Interaction of this hormone with cells in various organs results in upregulation in the expression of genes required for development and maturation of reticulocytes into erythrocytes, and production of other regulatory factors supportive of erythropoiesis, including those that enhance iron transport to the bone marrow after its mobilization from ferritin or absorption from the diet. Major players regulating the iron transport aspect include the small peptide hormone hepcidin produced mainly by liver hepatocytes, and the iron efflux transporter ferroportin, with which it interacts in the plasma membranes of intestinal mucosal cells as well as those of macrophages and hepatocytes in the liver and spleen. Our current understanding of the steps and mechanisms for mobilizing the iron stored in ferritin in support of erythropoiesis in the bone marrow and/or formation of iron enzymes and cytochromes within cells is the subject of this review.

## 2. Ferritin Structure and Formation for Iron Storage and Detoxification

Ferritin is a large hollow, symmetrical protein, usually comprised of mixtures of two kinds of paired homologous but not identical subunits (heavy and light; H and L; about 20 kDa) expressed from separate genes, for a total 24 subunits and a molecular weight of about 480 kDa [7,8,9]. In cardiac and skeletal muscle, a larger form comprised of 36 subunits is also present [10,11,12]; and “mini” ferritins with 12 subunits are produced by bacteria [13,14]. In mammals, most ferritin is produced by cytosolic ribosomes and found in that cellular compartment [15], although some is also found in lysosomes (see later), and some is secreted into the extracellular fluid and blood plasma [16,17,18] after synthesis on ER-bound polyribosomes. Mitochondria also contain some ferritin (24-subunit, H-like) encoded by a separate, intronless nuclear gene [19]. Like other ferritins, it sequesters iron to supply the metal for heme biosynthesis (a major function of mitochondria) and perhaps to protect mitochondria against iron-enhanced ROS production [20]. (It is not the subject of this review). X-ray structural analyses of the main (non-mitochondrial) ferritin indicate that the hollow protein “shell” is composed of pairs of subunits (associated head to foot) arranged with 432 symmetry, so that pores of two kinds are produced: hydrophilic ones where portions of three subunits combine, and hydrophobic ones made up of portions of four subunits. The wider hydrophilic pores (eight per ferritin molecule) are involved in uptake and deposition of ferric iron within the structure. The function (if any) of the hydrophobic pores is unknown [21]. The inside of ferritin is where iron not currently being transported or utilized is stored in the form of ferrihydrite crystals (5Fe_2_–9H_2_O) [22]. Storage is very efficient. It has been estimated that up to 4500 atoms of Fe can be accommodated within a single 24 subunit molecule, although the average Fe content (at least in liver and spleen cells) tends to be about half of that or less [23,24]. Ferritin from non-hepatic tissues, as well as cancers, tend to have a lower iron content [25,26]. The iron content of individual ferritin molecules covers a wide range; some molecules have much and others little or no iron. This has been determined by electron microscopy, where the relative amounts of electron-dense iron crystallites within donut-like ferritin shells can be viewed directly and by separating batches of purified ferritin in density gradients, where density depends upon the iron content, and measuring the iron:protein ratio [8,27,28,29,30]. Deposition of ferrihydrite crystals in ferritin occurs *in vitro* upon incubation of the protein with Fe^2+^ in the presence of oxygen [29,31]. This process involves movement and oxidation of Fe^2+^ and reduction of oxygen (ferroxidation) by several means and in several stages. Stages include entry of Fe^2+^ into the three-fold channels of the ferritin “shell”; oxidation to Fe^3+^ at ferroxidase active sites of H subunits involved with these channels [31]; transfer of Fe^3+^ (and some Fe^2+^) to the interior; binding to nucleation sites (glutamate and histidine residues on L subunits) within the cavity that promote formation of microcrystals, mainly of ferrihydrite; and additional Fe^2+^ oxidation on the surface of the microcrystals already deposited there [32]. It has plausibly been assumed that *in vivo* iron deposits within ferritin in the same manner as it does *in vitro*. However, some iron deposition may be aided by a recently reported cytosolic chaperone [33,34]. The large capacity of ferritin for iron and the fact that once converted to mineral the iron becomes innocuous and cannot participate in redox chemistry, renders ferritin not only an efficient means of storing excess iron rapidly but also an efficient means of detoxifying the metal.

Another aspect of ferritin metabolism supportive of detoxification is that synthesis of additional ferritin protein is stimulated by influx of iron into the cell cytosol [5,6]. Moreover, this system is mainly post-transcriptional, involving removal of a translational inhibitor (iron response proteins IRP1 or IRP2) bound to an iron response element (IRE) in the 5′untranslated region (UTR) of ferritin mRNA, resulting in an immediate burst of translation that leads to more ferritin. Thus, whenever iron enters the “labile iron pool” of the cell cytosol (such as from endosomes after receptor mediated endocytosis of transferrin, or after direct uptake through cell surface transporters like DMT1 and ZIP14 [35]) it finds ferritin molecules that still have lots of space to take in more iron. Simultaneously, more ferritin is made to ensure adequate numbers of ferritin molecules are there to take in more. The presence of this kind of dynamic and adaptive iron storage system underscores the need for cells to immediately store and detoxify any incoming iron not immediately utilized by iron-dependent proteins, so that oxidative damage is prevented or minimized. As a result, the iron status of a cell determines the rate at which ferritin is expressed, and its inherent ferritin iron and protein concentrations. This works both ways, so that when iron is lost or removed from the cell, ferritin iron and protein concentrations fall (see section 3.2 on mechanisms of ferritin degradation).

The cytosolic “labile iron pool” [36] is thus the major factor controlling levels of ferritin iron and protein. It represents iron fluxing in and out of cellular proteins and compartments and is not currently associated with iron-dependent enzymes, cytochromes, and oxygen carriers, or already stored in ferritin. Though of uncertain composition and defined as “chelatable iron”, the labile iron pool regulates not only the translation rate of ferritin mRNA but that of a whole series of other proteins involved in iron metabolism, including transferrin receptor 1. Transferrin receptor 1 mediates most of the cellular iron uptake, by binding Fe-transferrin at the cell surface, which is internalized by receptor-mediated endocytosis; release (by low pH) and reduction of the iron in endosomes (by a reductase, possibly Steap 3 [5,37], or endosomal ascorbate [38]); and transport of the released iron into the cytosol via divalent metal transporter 1 or other transporters. Other iron-related proteins controlled by the labile iron pool include reticulocyte δ-amino levulinate synthase, the rate-limiting step in porphyrin and heme biosynthesis that provides heme for hemoglobin, myoglobin, cytochromes and heme-dependent enzymes; and ferroportin, the major iron efflux transporter in the cell plasma membrane, which will also release iron from cells in storage organs (like liver and spleen) to the blood for transport to the bone marrow. Messenger RNAs for all these enzymes have iron response elements (IREs) in either the 5′ or 3′UTRs. As with ferritin mRNA, the 5′-UTR location of the IRE in δ-amino levulinate synthase mRNA allows iron response proteins, IRP1 or IRP2, to bind to the IREs and block translation. Since iron inactivates or reduces levels of the inhibitory IRP1 and 2, respectively, production of ferritin in all cells and heme in erythroid cells is positively coordinated with iron influx or availability, and *vice versa*. In contrast, multiple IREs in the 3′-UTR (as in transferrin receptor 1) allow for stabilization and accumulation of specific mRNAs through IRP binding, resulting in more mRNA to be translated, more protein, and in the case of transferrin receptor 1, uptake of more iron to remedy iron deficiency. A lack of IRP binding to those mRNAs results in mRNA destabilization and degradation, with the opposite overall effect. Thus, cells deficient in iron have IRPs bound to IREs in the 5′ and 3′-UTRs of several mRNAs, resulting in inhibition of production of some proteins (like ferritin and δ-amino levulinate synthase) and increased production of others, like transferrin receptor deployed on the cell surface to bring in extra iron. In contrast, cells in which iron is entering are producing ferritin to store it, and reducing their levels of transferrin receptor, since they are not lacking the element. In the long-term, there is additional regulation of expression of these iron-involved proteins at the transcriptional level through mechanisms not yet fully understood. Iron status also affects rates of transcription of ferritin genes in several cell types and organisms [39], increasing expression with long-term enhancement of iron availability. Hormonal regulation (particularly of ferritin H transcription) also occurs in some cells [40,41].

## 3. Release of Stored Iron from Ferritin

### 3.1. Regulation of Ferritin Degradation

While ferritin synthesis is stimulated by increased levels of iron in the cytosolic labile iron pool through removal of IRP inhibition of ferritin mRNA translation, a lowering of labile iron has the opposite effect. With less iron in the labile iron pool, most or all ferritin mRNA is bound to inhibitory IRPs that block translation, and thus, rates of ferritin synthesis are much lower or virtually nil, while some degradation of ferritin may still be occurring. However, the degree of IRP binding to ferritin mRNA (and thus, the rate of ferritin synthesis) is not the only factor determining levels of ferritin protein in cells at any given moment. Alterations in rates of ferritin protein degradation are also involved. Studies from our laboratory some time ago in cultured rat hepatoma cells showed that the levels of iron influx regulated the rates of ferritin protein degradation, as measured by loss of ^35^*S*-methionine/cysteine-labeled ferritin protein over time, and by other methods [42]. In fact, these studies showed that high levels of iron influx (exposure to 180 µM ferric ammonium citrate) virtually eliminated degradation of ferritin protein, while also stimulating production. In contrast, prevention of further iron influx by treatment of cells with the iron chelator, desferrioxamine (DFO) [42,43], or equally well by transfer of cells to a low-iron medium [42], resulted in ferritin degradation at rates that varied with cell type (half-lives ranging from 11 to 23 h). This was observed in models for enterocytes (Caco2 cells), hepatocytes (rat hepatoma cells H4-II-E-C3), and reticulocytes (K562 cells differentiated with tributyrin), each of which handle iron in very different ways. Enterocytes (and Caco2 cell monolayers) are involved in regulating dietary iron absorption. They take up less iron across their apical brush border and release less to the blood side when iron is abundant; and take up more dietary iron and release more to the blood when iron is relatively unavailable. (It is interesting that Caco2 cell monolayers with tight junctions display the same, seemingly selfless trait as enterocytes do *in vivo*, releasing most of the iron they absorb to the basolateral chamber, representative of the blood, in response to iron deprivation [44,45]). Hepatocytes are particularly important for storage of extra iron that can be called upon when needed. They respond to iron influx by accumulating ferritin iron and protein and lose ferritin iron and ferritin protein when iron influx ceases. Reticulocytes are focused on incorporating iron into hemoglobin and differentiating into finished erythrocytes that carry oxygen in the circulation. Good iron availability in the blood results in rapid uptake from Fe_2_-transferrin by receptor mediated endocytosis, and the iron is incorporated into the porphyrin ring to form heme for hemoglobin (through ferrochelatase in mitochondria) or into ferritin for temporary storage. Less circulating iron-replete transferrin results in less iron uptake and lowered rates of synthesis of porphyrin/heme as well as of the globin portion of hemoglobin [46]. Thus, iron regulates not only the rate of synthesis of ferritin but also its rate of degradation [42]. Exactly how iron regulates ferritin degradation however is unclear and has not been well studied. However, Bridges and Hoffman showed that ascorbate (1 nmol/10^7^ cells) reduced rates of degradation and iron release [47].

The results of more recent studies in some other cell types suggest that iron may not always be regulating rates of ferritin degradation. Asano *et al*. [48] found that in cancerous HeLa cells, iron deprivation enhanced degradation of ferritin compared to that occurring in the presence of iron in the medium, but that in primary and cultured fibroblasts ferritin protein was turning over at about the same rate whether in the presence of 100 µM ferric ammonium citrate (FAC) or in a state of iron deprivation. Degradation was also measured by following loss of ^35^*S*-radioactivity from labeled ferritin in cells exposed to FAC or made iron deficient by exposure to DFO, which enters lysosomes, or with another chelator (bathophenanthroline disulfonate) that stays outside the cell. (Both forms of degradation appeared to involve the lysosome, but in different ways—see later.) The authors suggest that continuous turnover of iron-containing ferritin might be a normal process, while cancer cells might gain protection against iron-dependent formation of reactive oxygen species (ROS) by only degrading ferritin when its iron is needed for cell proliferation. Consistent with their hypothesis, the hepatic cells in which we demonstrated cessation of ferritin degradation when culturing in 180 µM ferric ammonium citrate were cancerous (a hepatoma cell line). However, other explanations are also possible, including that these processes vary with cell type, and that differences in rates and mechanisms of iron uptake might result in different steady-state levels of the labile iron pool in the cytosol, which may be the key to iron regulation of ferritin turnover.

### 3.2. Mechanisms of Ferritin Degradation and Release of Its Iron

In theory, there are two mechanisms by which ferritin protein, like other proteins, might be degraded. These are through lysosomal proteolysis following autophagy from the cytosol into lysosomes, and by the proteasome in the cytosol following tagging by ubiquitin. Evidence for the involvement of both processes has been reported. The relative importance of these two degradative routes for ferritin and their consequences may vary among cell types. What we know about these processes is here described.

#### 3.2.1. Lysosomal Ferritin Degradation

It has been known for a long time that while most ferritin tends to be in the cytosol where it is produced, some is located in lysosomes, especially in iron loaded cells [49,50,51,52]. Moreover, a number of different investigators have obtained solid evidence that in a variety of cell types, lysosomal proteases are involved in ferritin degradation and release its iron, and indeed that this may be the main route for ferritin degradation and iron release. Lysosomes are a cellular compartment that is constantly in flux. It consists of fusing and dividing vacuoles, into which degradative enzymes flow from the Trans Golgi Network, and substrates to be degraded enter via autophagy of intracellular components (and organelles), or by fusion with endocytic vesicles containing extracellular or plasma membrane materials [53]. Lysosomal pH (5–5.5) is optimal for the activity of its cysteine, aspartate and serine-type proteases (cathepsins) of which there may be at least 14 varieties [54]. Lysosomes also contain high concentrations of thiol reducing agents, particularly glutathione and cysteine [55,56] but also ascorbate. These have the potential to react with the iron, including that in ferritin iron crystallites (see later). Apart from the role of lysosomes in degrading materials—such as aged red cells or receptor-hormone complexes from the plasma membrane (all endocytosed from the environment)—they also degrade and recycle internal membrane materials, and proteins—particularly those with long half-lives—which include the enzymes of the proteasome [57,58]. Overall, ferritin would seem to fit into the category of long-life proteins, those with half-lives of days rather than hours. For example, we reported the half-life of ferritin protein in rat liver to be about 2.5 days [24]. However, in cell culture, the half-life can be as short as 3.5 h [59].

Bridges and colleagues [47,60,61] were among the first to provide evidence that ferritin entered lysosomes for its degradation. Using erythroleukemic K562 cells they found that ferritin was autophagized by lysosomes. Moreover, ferritin autophagy and degradation were inhibited by ascorbate, and this prevented ferritin iron release, implying that lysosomal proteolysis was needed for mobilization of its iron. Subsequent studies by Konijn and others [62,63] using erythroleukemic K562 cells as well as primary human erythroid cultures showed that inhibition of lysosomal proteolysis by specific inhibitors like leupeptin and chymostatin, or raising the lysosomal pH with chloroquine, reduced the rate of transfer of ^59^Fe tracer from ferritin to hemoglobin, and prevented the concomitant decrease in ferritin protein normally observed during reticulocyte development. (In this case, iron deprivation resulting in ferritin movement to lysosomes was initiated with the SIH, a cell permeant iron chelator that accumulates mainly in the cytosol). Radisky and Kaplan [64] showed in fibroblasts that endogenous as well as exogenously administered (cationic) ferritin was degraded by lysosomes, and that the resulting iron released returned to the cytosol, where it induced the formation of (and was incorporated into) new ferritin. Ollinger and Roberg [65] reported the same phenomenon for rat hepatocytes, namely that as a consequence of iron lack (through serum deprivation or treatment with the strong chelator desferrioxamine; DFO), cytosolic ferritin moved to lysosomes and this increased iron levels of the cytosolic labile iron pool. As already mentioned, we reported in 2001 [42] that for rat hepatoma cells, rates of ferritin degradation and ferritin iron release were parallel processes, implying that ferritin protein degradation was required for release of its iron. Larson *et al*. [66] confirmed in 2004 that ascorbate prevented ferritin redistribution to lysosomes, lysosomal ferritin degradation, and ferritin iron release induced by infection of epithelial cells with the pathogenic bacterium *Neisseria meningitidis*, which needs the iron for proliferation. Kwok and Richardson [67] demonstrated in cardiac and cancer cells (including melanocytes) that lysosomal protease inhibitors, including doxorubicin, promoted accumulation of ferritin iron intracellularly. We then reported that the same kinds of treatments (leupeptin, chymostatin, chloroquine) reduced rates of ferritin iron release and ferritin protein degradation in enterocyte, hepatocyte and reticulocyte cell culture models [43]. In these studies, cells were first treated to induce production of ^59^Fe-labeled ferritin and then deprived of iron by reculturing them in the presence of DFO, or exposing them to a culture medium low in iron without the presence of an iron chelator. Iron deprivation resulted in first order losses of ferritin protein and ferritin iron over time, with half lives in the range of 10–25 h. It was noteworthy that again the time course for loss of ferritin iron was identical to that for loss of ferritin protein, implying that both processes were occurring simultaneously, and that one was dependent upon the other. (This agreed with conclusions from our earlier studies of the regulation of ferritin degradation by iron [42].) Another observation was that in these cells, inhibitors of proteasomal degradation (lactacystin or MG132) did not alter ferritin degradation or iron release [43].

Much more recent reports confirm and extend these observations. Zhang *et al*. [68] reported that in a mouse B cell hybridoma cell line, lysosomal proteolysis was the primary degradation pathway for cytosolic ferritin, and that degradation of ferritin protein by lysosomes was necessary for release of its iron. In this case, ferritin iron degradation was triggered by overexpression of mitochondrial ferritin, which preferentially took up the iron released from cytosolic ferritin after lysosomal degradation. Initiating expression of mitochondrial ferritin (repressed by tetracycline) triggered degradation of cytosolic ferritin and increased its colocalization with a marker (LC3) of autophagosomes (indicative of macroautophagy—see later). Cytosolic (and mitochondrial) ferritin responses were monitored by immunoblotting, but also by labeling with ^35^*S*-amino acids and ^59^Fe. Lysosomal protease inhibitors (leupeptin and E64D) but not an inhibitor of the proteasome (lactacystin) inhibited degradation of ferritin protein and release of its iron but did not affect deployment of transferrin receptor on the plasma membrane and the capacity for iron uptake. Curiously, they also presented some evidence that blocking proteasomal degradation with lactacystin promoted stabilization of cytosolic ferritin when cells were kept in a high iron environment. This finding is discussed further in the section on ferritin degradation by proteasomes (below).

A major recent study confirming the importance of lysosomes is that of Asano *et al*. [48], in which degradation of ferritin was studied in primary and transformed mouse embryonic fibroblasts. In these studies, degradation was monitored in ferritin radiolabeled with ^35^*S*-amino acids. Turnover caused by DFO, the non-endocytosed iron chelator bathophenanthroline sulfonate, or by activation of ferroportin expression (enhancing iron efflux) was lowered by treatment with (a) inhibitors of lysosomal proteases (E64D and pepstatin); (b) an inhibitor of lysosomal acidification (bafilomycin A, which prevents proton influx); and (c) decreasing expression of proteinaceous factors that mediate macroautophagy of proteins into lysosomes (ATG5 and ATG7). Degradation rates were not affected by inhibition of the proteasome (lactacystin). Although release of iron from ferritin was not directly monitored, reduced lysosomal function resulted in lysosomal iron accumulation (as monitored by transmission electron microscopy-energy-dispersive X-ray analysis) as well as in cytosolic iron deficiency (monitored by IRP binding). Moreover, inhibition of lysosomal acidification (with bafilomycin A) had more of an effect on retention of ferritin iron by lysosomes than did treatment with pepstatin and E64d. This makes sense in that (a) the protease inhibitors used may not have decreased the activity of all the proteases involved in ferritin degradation; (b) increasing lysosomal pH would decrease the effectiveness of all the lysosomal proteases, slowing the degradation of the ferritin protein “shell”; (c) the decreased acidity would most likely also reduce the solubilization of the ferrihydrite crystallites of ferritin upon their exposure to the lysosomal environment (see more in section 3.4 below). Together, all of these studies clearly show that in a variety of different cell types, mobilization of the iron stored in ferritin is at least in large partly dependent on its movement into lysosomes (via macroautophagy) and degradation of its protein “shell” within that organelle. Whether and how the cytosolic proteasome system may be involved is discussed in the next section. In these same cell types, inhibitors of the proteasome were not effective in preventing ferritin iron release.

Another observation already mentioned and worth emphasizing [48] is that at least in embryonic fibroblasts, ferritin protein turned over even under conditions in which cells were continuously exposed to 100 µM of ferric ammonium citrate. Moreover, degradation was still mediated by lysosomal proteolysis, but through micro- rather than macro-autophagy (see more about these distinctions below); and the authors provided preliminary evidence that cancer cells might differ from non-cancer cells in only degrading ferritin in response to iron deprivation, e.g., via macro-autophagy, which might render them more resistant to iron toxicity. (Ferritin would only be turning over and releasing iron when it was needed for cell growth).

A large body of additional evidence has accumulated indicating that lysosomes are the major site in cells where redox active iron is released through degradation of hemoglobin, ferritin and other iron proteins. Many of these studies are from the group of Brunk and Kurz, who have concentrated on the potential importance of this organelle-confined process in shielding other cell parts from potential oxidative damage that iron might otherwise engender [53,69]. Since it is well known that iron ions released from proteins can undergo Fenton chemistry to produce damaging reactive oxygen species (ROS), confining this chemistry mainly to lysosomes would spare more vital portions of the cell. They point out that Fenton chemistry proceeds more rapidly at lower pH, as in lysosomes; and that lysosomes are the most likely to contain ROS damaged molecules such as are present in lipofuscin granules [53,56,69], which are aggregates of chemically-altered, oxidized, and no longer degradable lipid, protein and/or polysaccharide. These granules have long been considered related to induction of cellular aging and senescence [70]. Kurz *et al*. [53] contend that lysosomes contain most of the labile iron in cells. In support of this, they showed that partial permeabilization of lysosomal membranes in cultured macrophages (J774 and THP-1) by a specific detergent, induced ferritin production in the cytosol, presumably due to release of lysosomal iron. Alkalization of lysosomes with ammonium chloride prevented this detergent-dependent ferritin induction, consistent with the concept that degradation of ferritin and other iron proteins (which would be much slower at alkaline pH) provided the lysosomal iron that induced ferritin production [71]. Interestingly, they also reported that some inhibitors of cathepsins B and L did not have the same inhibitory effect as alkalization. This implies that other cathepsins are responsible for ferritin proteolysis in macrophage lysosomes, although cathepsin B (inhibited by leupeptin) is implicated in ferritin degradation in many other cell types (see earlier). Another possibility is that alkalization itself made lysosomal iron unavailable, e.g., unable to induce ferritin synthesis by removing IRPs from its mRNA.

Kurz, Brunk and colleagues [53,69,72] propose that while lysosomes are more vulnerable to ROS damage due to their role in degrading ferritin and other iron-containing proteins, their low pH and reducing environment, mechanisms to reduce ROS formation in lysosomes have also evolved. These would include a rapid return of iron to the cytosol (for incorporation into and detoxification by ferritin) and temporary binding to protective proteins. Specific proteins brought into lysosomes would mitigate ROS production. They have accumulated direct and indirect evidence that ferritin (and particularly apoferritin), metallothionein (MT), and heat shock protein 70 (Hsp70) reduce ROS production in lysosomes, and point out that expression of all three is induced by oxidative stress [40,41,73,74]. Overexpression of the chaperone Hsp70 protected lysosomes against oxidative stress [75]; *in vitro* at lysosomal pH, pure Hsp70 (0.5–1.5 µM) very effectively reduced ROS production, in contrast to the pure albumin control (1 µM) [76]. To generate ROS, they mixed 10 µM FeCl_3_, 100 µM cysteine and 100 µM peroxide, in the presence of dihydro-dichloro-fluorescein, and monitored changes in fluorescence. As concerns metallothionein (MT), they cite studies of others [73] that oxidative stress increases expression of MT in liver, and that a high expression increases resistance to oxidative stress. In Kurz and Brunk’s own studies [77], high levels of MT induced by zinc treatment of cultured macrophages (J774) reduced apoptosis and oxidative damage to lysosomes. Here, they introduced iron into lysosomes by treating with FeCl_3_ which they claim forms an endocytosed iron-phosphate complex that can be seen within the lysosomes by electron microscopy after treatment by the sulfide-silver method. Cells were treated with the fluor dihydro-dichloro-fluorescein that enters the lysosomes. ROS formation was then induced by peroxide. If lysosomes were damaged, the fluor leaked into the cytosol and fluoresced in response to the increased pH of its environment. They also showed that at least *in vitro*, MT binds iron (presumably Fe^2+^). A significant caveat however is that, although they state that both MT and Hsp70 might be authophagized into lysosomes, they did not actually provide evidence for this being the case.

Their work with ferritin is more extensive and concrete. In several studies, they showed that extracellular apoferritin introduced into macrophage lysosomes via endocytosis protected lysosomes against oxidative damage [50,53,69,78]. Introduction of iron-rich ferritin was less effective than apoferritin, but still provided some protection. Others have shown that H-ferritin may be particularly helpful in protecting whole cells against oxidative stress [79,80]. However, that may simply reflect the ability of ferritin (and particularly H-ferritin) to oxidize Fe^2+^ and sequester it in a way that does not damage cells. Overall, these various studies indicate that mobilizing stored iron in lysosomes, after ferritin autophagy, is not only a major function of the lysosomal compartment but that during solution of the iron within ferritin, the un-degraded or partially degraded ferritin still present (and perhaps also some other proteins, like MT and Hsp70), is/are protecting the lysosome against ROS production and damage.

Ferritin autophagy and its triggers have not been well studied. Autophagy of cytosolic components into lysosomes can occur in several different ways. There are at least three ways for cytosolic proteins to enter lysosomes for degradation, macro-autophagy, micro-autophagy, and autophagy assisted by chaperone proteins [81]. In macroautophagy, small portions of the cytosol (containing a variety of substituents) become enclosed by a double membrane to form autophagosomes that eventually coalesce with lysosomes for degradation of their contents [82,83]. Microautophagy is a kind of endocytosis involving invagination of the lysosomal (rather than plasma) membrane [81]. Autophagy via chaperone proteins is a burgeoning field of investigation, since malfunction of factors involved in these processes are implicated in various diseases involving protein aggregates [81,84,85]. “Chaperone mediated autophagy” (CMA) is the best-known chaperone system, and mainly targets proteins that have amino acid sequences approximating KFERQ (lys, phe, glu, arg, gln) in their structure. Immunoprecipitation studies with KFERQ antibodies indicate that about 30% of cytosolic proteins may be in that category. For import into lysosomes, a chaperone Hsc70 (formerly known as Hcp73: heat shock cognate protein 73) binds to the KFERQ sequence in an ATP hydrolysis dependent reaction. Then, with the help of co-chaperones (including Hsp90, Hsp 40, and BAG1), the complex interacts with its lysosome receptor, lysosome-associated membrane protein 2 (LAMP2), unfolding the target protein and threading it into the lysosome through the LAMP2 channel [81,86,87]. A chaperone-assisted “selective” autophagy process is also recognized, which can (like for the proteasome) involve tagging with ubiquitin (see below). However, this tag leads to lipidation of the protein and entry into lysosomes [85,88,89]. Chaperone-associated ubiquitin-ligase (CHIP) assists in this form of autophagy [88]. Possibly, only large misfolded proteins that cannot easily enter the proteasome are targeted this way [88]. Ferritin, though not normally misfolded, is very large, and might also in theory have trouble entering the proteasome, although oxidized ferritin is efficiently degraded at least by the 20S proteasome (without the 19S “lids”), as demonstrated *in vitro* (see below). Ferritin can also form natural aggregates, and protein aggregates are often the target of lysosomal autophagy and can even inhibit the activity of the proteasome/ubiquitin-tagged degradation system in the cytosol [37,90] (see more below).

The mechanisms of ferritin’s autophagic entry into lysosomes are only just beginning to emerge. It was already noted some time ago by Bridges *et al*. that ascorbate inhibited ferritin autophagy [47,60,61]. How (or if) autophagy *per se* might be regulated by ascorbate is unknown. Since ascorbate would reduce Fe^3+^, ferrous iron might be part of the signal. As already indicated, recent work of Zhang *et al*. [68] with mouse B cell hybridomas, and Asano *et al*. [48] with embryonic fibroblasts both provided evidence that macroautophagy is involved. Knockout or knockdown of factors associated with formation of autophagosomes [68] and other aspects of macroautophagy (ATG5 and ATG7) [48] inhibited ferritin turnover induced by iron deprivation. However, in the latter studies with fibroblasts, ferritin also turned over in the presence of high levels of iron in the medium, and this mechanism of degradation (also dependent on lysosomes) was not affected by knocking out ATG5 and ATG7 It also was not prevented by knocking out LAMP2, one of the factors involved in the CMA form of chaperone-mediated autophagy. This implies that two kinds of autophagy might bring ferritin into lysosomes, depending on circumstances and cell type. Preliminary studies of a similar kind in a fibroblast line were carried out by the group of Jerry Kaplan and Ivana De Domenico [91]. Here as well, deletion of LAMP2 (and LAMP1) had no effect on ferritin degradation induced by iron deprivation with the chelator DFO, further supporting the idea that CMA is not involved in ferritin autophagy, although they indicated that ferritin does contain some KFERQ-like motifs. These investigators also reported that the classic inhibitor of autophagosome formation (macroautophagy) 3-methyladenine [92] failed to inhibit lysosomal ferritin degradation in HEK293T cells; but this contrasts with the findings of Asano *et al*. [48] for fibroblasts, who found it did inhibit the lysosomal degradation of ferritin that took place in the presence of 100 µM iron in the medium. This latter form of lysosomal ferritin autophagy (also thought to be macroautophagy), however, was distinguished from the macroautophagy occurring in response to iron deprivation, in that it was not dependent upon ATG5 and ATG7. Meanwhile, it appears that the actions of 3-methyladenine are much more complex than originally thought, making conclusions from its use less certain. Studies of Wu *et al*. [93] indicate that 3-methyladenine can both promote and inhibit autophagic flux depending upon the nutritional composition of the culture medium, and that this happens through differential effects on phosphatidylinositol-3-kinases (PI3K Classes I and III) and resulting signaling pathways. Overall, the data so far suggest that for ferritin, at least two kinds of lysosomal autophagy that include macroautophagy but not CMA may be occurring under different circumstances and in different cell types. Whether these findings can be confirmed and extended to other cells remains to be seen.

A relatively unknown fact about ferritin that might be related to its mechanism of autophagy is that it does form aggregates. Since, as already mentioned, protein aggregation can trigger autophagy [90,94], formation of ferritin aggregates might perhaps be a preliminary step leading to lysosomal uptake. The presence of ferritin aggregates, also known as ferritin oligomers, was established long ago, using electron microscopy of purified ferritin first separated in native PAGE [30,95,96,97]. Ferritin preparations were found to contain covalently-linked dimers, trimers, tetramers, and larger oligomers, usually representing no more than 10% of the total ferritin. (Much larger array-like aggregates of partially degraded ferritin—known as hemosiderin—also occur [30], particularly in iron overload). The way in which ferritin oligomers are formed and their purpose (if any) within cells is unclear. It has been demonstrated that individual ferritin molecules can become linked through disulfide bonds between external cysteines when oxidation is induced by loading iron into ferritin *in vitro*, which is also prevented by mercaptoethanol and the radical scavenger ceruloplasmin [98,99]. However, the original studies of the naturally occurring oligomers showed that treatment with disulfide reducing agents did not disassemble them [97]. So, oxidation is not likely to be involved in their formation. More recently, Hasan *et al*. [100,101] obtained *in vitro* evidence that ferritin aggregates while binding to tubulin fibers, and that this also occurs *in vivo* in the cytosol of several cell types. Moreover, they presented *in vitro* evidence that the fraction binding to tubulin (and aggregating) was 2.5-times richer in iron than the ferritin as a whole. If this is the mechanism of its oligomerization, and ferritin oligomerization also induces autophagy into lysosomes, then the ferritin entering lysosomes must be particularly iron rich, which seems possible.

#### 3.2.2. Proteasomal Degradation of Ferritin

There are some reports that proteasomal degradation of ferritin can occur. Proteasomes are very large dumb-bell-like structures in the cytosol, with an internal hollow proteolytic (20S) cylinder or barrel. They particularly dispose of proteins with short half-lives and/or those that have PEST segments (rich in pro, glu, ser and thr residues) [102]. They also degrade oxidized or otherwise damaged, misfolded or mutant proteins. Proteins are degraded after being unfolded and entering the internal hollow barrel (the “20S proteasome”) made up of arrays of several different proteases, sealed at either end by bulky “lids” (19S) to form the overall 26S proteasome. Most proteins targeted for proteasomal degradation are tagged with chains of ubiquitin (Ub), a 76 amino acid protein that is one of the most conserved and abundant in nature. Tags of at least 4 (and up to 50) Ub monomers, linked to each other and to their target proteins via a peptide bond between the C-terminal carboxyl group of Ub and a lysine-amino group (lys 48 in Ub), cause the proteasome to identify and degrade the tagged proteins. Ub-protein ligases (E3 enzymes) (of which there are many) recognize specific target proteins as substrates for polyubiquitination. Tagged proteins bind to the “lids” of the proteasome, which remove the poly-Ub, and unfold and release the target into the barrel for degradation with the help of ATP hydrolysis [103]. However, degradation of damaged/oxidized proteins appears to occur by a mechanism not involving Ub or ATP [104] (see more below).

Reports dating back to 1994 indicate that oxidized ferritin can be degraded by proteasomes [104,105,106]. Rudeck *et al*. [106] showed *in vitro*, using the purified 20S proteasome (and no ATP or Ub ligation system), that pure ferritin oxidized by a variety of agents was degraded three to four-times faster than the native form. It should be noted that some degradation by the proteasome occurred even with unoxidized (native) ferritin, at least in this *in vitro* system. Oxidation of the ferritin also resulted in release of some of its iron. However, it was interesting that proteolysis (by the proteasome) did not add to the iron released, from which they concluded that “the proteasome recognizes only ferritin molecules that are already depleted of iron, which are nonfunctional”. This is the first hint that iron-poor ferritin or apoferritin might be degraded by that route and by a system not involving Ub. Evidence for non-Ub-based proteasomal degradation of oxidized proteins and ferritin was then obtained by Shringarpure *et al*. [104] *in vitro* and *in vivo*, using Chinese hamster lung fibroblast cell lines and their lysates that did and did not express the Ub-activating (E1) enzyme required for target protein ubiquitination. In line with *in vitro* findings [106], oxidized proteins were degraded three to four-times faster than native proteins *in vivo* [104]. (Shringarpure *et al*. [104] did not test for ferritin degradation.) Rates of oxidized protein proteolysis were reduced by an inhibitor of the proteasome (NLVS) and only very mildly affected by the absence of ubiquitination. The potential effects of ubiquitination on degradation of oxidized ferritin by the proteasome *in vitro* were also tested. Oxidation by peroxide enhanced ferritin degradation. However, the presence of a ubiquitination system that included ATP, Ub and cell lysate made no difference. It did seem that some Ub had been added to the oxidized ferritin; however, the gels presented are somewhat hard to interpret. Following ferritin *in vivo*, Mehlhase *et al*. [14] then reported that MG132 (a well-known inhibitor of the proteasome) markedly reduced the rate of oxidized ferritin degradation induced by peroxide treatment of RAW264.7 macrophages, as monitored by the radioactivity remaining in ^35^*S*-labeled ferritin H and ferritin L subunits. (They did not examine turnover of unoxidized ferritin.) More recently, Zhang *et al*. [68] reported that while the primary site for ferritin degradation and release of its iron was the lysosome, the proteasome might also sometimes be involved. Using a B cell hybridoma line (B9) they confirmed that treatment with iron (as ferric ammonium citrate; FAC) stabilized cytosolic ferritin (measured by immunoblotting); that addition of leupeptin increased retention of ferritin protein about 20% over that of FAC alone; but that lactacystin (which inhibits the proteasome) also slightly enhanced ferritin stability (about 13%). Although this was a single study involving three “experiments” and the differences were small, the authors concluded that the proteasome could have a role in ferritin degradation under certain circumstances. Some more recent studies by the group of Jerry Kaplan and Ivana De Domenico [91,107] indicate that in addition to lysosomes, the proteasome can degrade ferritin, and particularly iron-poor or apoferritin. In 2006, they reported on studies in ferroportin-transfected HEK293T cells (under ecdysone control), in which they first produced ferritin by iron loading and then induced its degradation by activating ferroportin expression, which would promote efflux of iron from the cells [107]. Using this somewhat artificial system, they reported that the proteasome inhibitor MG132, or inactivation of the E1 ligase involved in ubiquitination, markedly reduced the loss of ferritin protein induced by ferroportin activation. However, this treatment did not influence the amount of ferritin iron lost. Although the iron content of the ferritin present in these cells was not actually measured (loss of ferritin iron being gauged by radiolabeling), these observations imply that iron-poor or apoferritin is being degraded by the proteasome but that iron-containing ferritin is not. In further studies with the same cell system, the group reported that degradation of ferritin could occur in both lysosomes and the proteasome, depending upon the chelator used for iron deprivation: DFO, which enters cells only by endocytosis [28], induced degradation of ferritin in lysosomes that was inhibited by chloroquine, whereas treatment with the permeant chelators deferriprone and desferasirox—that probably access iron in the cytosolic labile iron pool—resulted in ferritin degradation via the proteasome, being inhibited by MG132 and not chloroquine [91]. The finding that DFO is somehow unique among chelators in inducing migration of ferritin into lysosomes is not consistent with the findings of many others in more commonly used cell types (already cited) showing that simply switching to a low iron medium has exactly the same effect of inducing lysosomal ferritin degradation inhibited by chloroquine and inhibitors of lysosomal proteases [43,53,69].

### 3.3. Does Iron Exit Ferritin without the Protein First Being Degraded?

It has been known for a long time that iron can come out of ferritin *in vitro* without markedly disturbing its overall structure [8,27,30]. For this, ferritin isolated from organisms or tissues is usually incubated with strong reducing and chelating agents, such as mercaptoacetic (thioglycolic acid) acid and α,α′-dipyridyl, at pH 5, which results in formation of dialyzable Fe^2+^-chelates that diffuse out of the ferritin, through the hydrophilic threefold channels of the protein “shell”. Chelators like DFO that cannot enter the channels [108] can also receive the Fe^2+^ released from ferritin outside the protein [11] indicating that amino acids within the pore/channel are mediating release, just as they mediate entry. This is how iron is removed to make apoferritin *in vitro*, which can then for example be re-loaded with specific numbers of iron atoms in the presence of Fe^2+^ and oxygen, to study the mechanisms of ferritin iron entry and deposition/crystallization [28,29,32]. In recent years, studies of this sort (including those from the laboratories of Elizabeth Theil and Dennis Chasteen have increased our understanding of the mechanisms and steps by which iron is oxidized and internalized by ferritins with different proportion of H and L subunits, or upon mutating specific amino acid residues within channels and the interior cavity of ferritin [29,31,32].

Based on these and other studies (to be described), Theil sees ferritin as a nanocage with flexible iron pores (the hydrophilic channels) [21]. Moreover, she proposes that the pores resemble channels of transmembrane proteins through which cations diffuse, and that the pores are “gated” by specific bulky amino acid side chains that intrude into the ferritin channels [109]. More importantly, she sees the pores as serving not only for entry but also for exit of the iron. This clearly happens *in vitro*, and her laboratory has shown that certain mutations in amino acid sequence and/or exposure of ferritin to low concentrations of chaotropic agents like urea can enhance rates of exit. Some of these studies were motivated by the identification of a spontaneous mutation in recombinant frog H ferritin that resulted in a markedly faster rate of iron release by reducing and chelating agents [110].

To measure effects of various factors on iron release from ferritin, the pure protein (usually frog H ferritin) denuded of iron and reloaded with specific numbers of iron atoms per molecule, was typically incubated with NADH, FMN and bipyridyl in MOPS-NaCl, at pH 7, and the rate of Fe^2+^-bipyridyl complex formation was monitored over time [21,110]. Substituting proline for leucine 134, close to one end of the hydrophilic channels, resulted in an enlargement of these pores [21,111]. Substituting proline, valine, alanine or glycine for leucine 134, or making similar substitutions for leucine 110 (more central to the channel) resulted in three to four-times higher initial rates of iron release compared to the wild type, the proline substitution being the most potent [110]. Times for release of 80% of the ferritin iron were reduced to 8–24 min from about 60 min for the wild type, and that for the proline mutant (L134P) to 2 min. Altering the aspartate-arginine (ion pair) interaction (at the other end of the channel from leucine 134) had similar effects, and the same was the case with certain modifications of the loops between helices C/D and B/C. Theil’s group has also demonstrated that 1 and 10 mM concentrations of chaotropes (urea and guanidine HCl) enhanced rates of iron release [28]. These concentrations were much lower than those needed to denature the ferritin structure (which is stable to 6 M urea) and might cause localized opening of channel obstructions, as demonstrated by changes in the melting temperature transitions of some helix subdomains. Low concentrations of urea—in the range of those used in the experiments—might occur in certain cells (particularly those of the liver and kidney) normally or in pathological states, and this might influence ferritin pore structure and accessibility. All of these studies indicate that mutations that increase the size of the hydrophilic channels in ferritin or environmental factors that unfold flexible areas leading to widening of the channels can greatly increase the rates at which the iron inside dissolves so it can diffuse out of the structure. Since the rate of reduction of the ferrihydrite is the rate-limiting step in this process, increasing the size, width and/or accessibility of the channels would be expected to increase the rate at which agents like reduced FMN are able to access the internal mineral.

Theil’s group also screened a combinatorial library of random heptapeptides for tight ferritin binding with the idea that small peptides might perturb access to the ferritin iron pores and/or modulate the size and width of the channels or their flexible “gates” [112]. One (hydrophobic) peptide was found that enhanced iron release rates six-times; another (with a high histidine and glycine content) reduced release rates to about half. Although there is no evidence these peptides occur in cells, it seems plausible that there might be peptides that interact with pore-regions of the ferritin structure and regulate access of either iron or reducing/chelating agents to channels leading into and out of the hollow cavity in ferritins.

These concepts of the mechanism by which iron is released from ferritin are very reasonable and are based on solid evidence obtained through studies of the purified protein. What is missing, however, is evidence that the processes defined *in vitro* also occur *in vivo*. Indeed, as already described, virtually all the *in vivo* evidence supports a very different chain of events, involving degradation of the protein “nanocage” prior to release of the iron inside. Indeed, it might be more disadvantageous to the organism to leave control of iron release to changes in levels of ascorbate and/or glutathione, for example that might not be under as tight control, leading to efflux not connected with iron needs and promoting oxidative stress. Also, Bridges *et al*. [47,60,61] showed repeatedly that treating cells with high doses of ascorbate, a highly effective iron reductant and chelator, failed to enhance ferritin iron release, an observation that our laboratory has confirmed and extended to observations with other reductants and chelators [113]. Moreover, ascorbate actually had the opposite effect (see section 3.2), inhibiting lysosomal autophagy and ferritin iron release. This effect was also recorded by Ollinger and Roberg [65] in hepatocytes and by Larson *et al*. [66] in epithelial cells in which ascorbate inhibited ferritin redistribution to, and degradation in, lysosomes induced by infection with the pathogenic bacterium *Neisseria meningitidis*, which needs iron for proliferation.

### 3.4. Solubilization of Ferritin Iron in Lysosomes

Although we have described the abundant evidence for iron being released from ferritin in lysosomes following or in parallel with degradation of its protein “shell”, the steps leading to solubilization of the ferritin ferrihydrite nanocrystals that would allow transport of iron back into the cytosol are currently mainly in the realm of speculation. It is clear (from data already described) that the iron released from ferritin in lysosomes returns to the cytosol, where it induces new ferritin synthesis [50,58] and contributes to the labile iron pool [48]. It is also clear that lysosomes normally contain high concentrations of reducing agents that would seem capable of dissolving ferritin iron, notably glutathione and ascorbate [53,56]. In as yet unpublished studies from our laboratory presented at meetings [114] we found that 1–10 mM concentrations of glutathione and ascorbate readily dissolved large portions of ferritin mineral that had been extracted and purified from rat liver ferritin, both at pH 5 and pH 7. Extracts of fluid from purified cell or tissue lysosomes were able to solubilize the iron as well. Since these agents would not only chelate but also reduce the iron ions in the mineral, their actions alone would most likely produce Fe^2+^ that is ready for transfer across the lysosomal membrane into the cytosol, via specific transporters (see next section). Lysosomal pH alone is not sufficiently acidic to dissolve the mineral rapidly, which also suggests that reduction of Fe^3+^ by a reductase enzyme is not likely to be involved.

### 3.5. Transport and Distribution of Iron after Release from Ferritin in Lysosomes

Once the iron in ferritin has been mobilized by dissolving it with endogenous reducing and chelating agents like glutathione and ascorbate, it must be conveyed back into the cytosol and from here directed to where it is needed in the cell or exported into the blood. The specific steps involved are still being worked out. One would expect that one or more transporters residing in the lysosomal membrane would export Fe^2+^ to the cytosol, where it would associate with as yet ill-defined components of the labile iron pool, and then find its way to other cell compartments or Fe-dependent enzymes or to ferroportin in the plasma membrane through which it would exit as Fe^2+^. Thereupon it would bind to transferrin in the blood plasma, after oxidation to Fe^3+^ either by transferrin itself [115] or by one or another of the multi-copper ferroxidases present in the plasma [116] or on the cell surface [84,117]. Transferrin would then deliver it to cells all over the organism but particularly to developing reticulocytes in the bone marrow, in support of normal or enhanced erythropoiesis.

Several potential and actual iron transporters have been reported to be associated with lysosomal membranes. The most likely to be involved in lysosomal iron efflux is divalent metal transporter 1 (DMT1/NRAMP2), which, as already described, is very active in dietary iron uptake by enterocytes across the brush border membrane and also releases iron from early endosomes in which transferrin-transferrin receptor complexes are cycling after endocytosis of Fe-transferrin from the blood plasma (see earlier). We and others have reported its colocalization with lysosomes [114,118]. At least in macrophages and neutrophils, a homologous protein to DMT1, natural resistance-associated macrophage protein 1 (NRAMP1), is located in late endosomes and phagolysosomes (that express LAMP1) [119], where it is thought to promote loss of iron (and perhaps also other metals) from vesicles containing phagocytosed microbial pathogens, who need iron for growth and proliferation. (Expression of NRAMP1 in CHO cells resulted in increased rates of transport of Fe^2+^ as well as Mn^2+^ [120].) Studies with Nramp1^−/−^ mice showed that spleens (and to a much lesser extent livers) retained larger than normal loads of iron, and that the effect was exacerbated by overloading the circulation with damaged red cells induced by treatment with phenylhydrazine [121]. The same group, studying macrophage erythrophagocytosis with RAW 264.7 cells showed that knockdown of DMT1 with siRNA or cells with lower (*versus* higher) levels of Nramp1 expression resulted in retention of extra iron [122]. Knockdown of DMT1 in the low Nramp1 expressing cells, however, had the largest negative effect. Up to 40% of the ^59^Fe from the phagocytosed erythrocytes was retained in the cells after 24 h compared with 13% in the control.

Another potential candidate is TRPML1, the dysfunction of which is associated with mucolipidosis type IV and severe anemia [121]. Expressed in most cells [123] and located mainly in late endosomes and lysosomes [124], it has been demonstrated to transport iron when transfected into HEK293 cells [125]. It was also reported that skin fibroblasts from patients with the disease had more iron in their lysosomes (estimated histologically by a modified sulfide-silver method after exposure of cells to Fe-dextran), and that these cells also had lower cytosolic iron (measured with fluor-quenching). However, others demonstrated that knocking down TRPML1 in HEK293 cells resulted in enlarged lysosomes with high levels of zinc (shown by confocal microscopy) but no differences in total cellular Fe [126]. There was also an accumulation of Zn in mucolipidosis patient fibroblasts; and TRPML1 knockout mice had a significant but small (~15%) increase in zinc but not in other metals in their brains (determined by ICP-MS). Whether this ion channel normally plays a role in iron transport across cell membranes thus remains unresolved. A member of the Zip family of zinc (and iron) transporters, ZIP8, was also considered a possible lysosomal transporter. However, Wang *et al*. [127] showed that when overexpressed in HEK 293T cells, it enhanced uptake of Fe^2+^ across the plasma membrane and was found not so much in lysosomal membranes but in that of the cell and of early endosomes. Moreover, transport of iron was very poor at lysosomal pH, all of which makes it unlikely that ZIP8 participates in iron efflux from lysosomes.

Once in the cytosol, the iron in the labile pool (presumably as Fe^2+^ because of the continued reducing environment) will conceivably be carried to other sites in the cell where it might be needed, or shuttled to the plasma membrane where it could be transferred to the blood plasma for transferrin transport in the circulation. Sites within the cell that might require iron would include the mitochondria, where iron is incorporated not only into Fe-S clusters for specific enzymes (including aconitase in the TCA cycle) but also into protoporphyrin IX by ferrochelatase, the last step in heme biosynthesis. Interestingly, endogenous cytosolic ferritin may not be a source of iron for heme synthesis in some cells, as reported by Mikhael *et al*. [128] for the cultured macrophage line RAW264.7 in which heme biosynthesis was stimulated by incubation with the 5-amino levulinate from which porphyrin is produced. How iron is shuttled from one place to another within the cell cytosol, and as part of the labile iron pool, is still uncertain but may involve potential chaperones of various kinds [5,33,34,77,129].

What is certain is that ferroportin in the plasma membrane is a major route by which iron is released by most cells into the blood. Moreover, it is the only as yet identified iron exporter [37]. Knocking out ferroportin expression results in a profound iron deficiency ascribable not only to reduced absorption of dietary iron (due to reduced efflux into the blood from enterocytes), but also to a reduction in cycling of iron in red cell hemoglobin that must be released from macrophages and hepatocytes and returned to bone marrow erythropoiesis [37,130]. Ferroportin knockout is embryonic lethal in mice; deletion *in utero* after initial embryogenesis results in pups that die soon after birth and show accumulation of iron in enterocytes, macrophages and hepatocytes, consistent with the importance of this exporter for dietary absorption and red cell iron cycling. Interestingly, knocking out 90% of ferroportin expression in macrophages led to only mild anemia [131]. This suggests either that the remaining 10% was sufficient to carry out the bulk of the iron efflux involved in red cell iron recycling, and/or that there are other iron efflux mechanisms. (Studies by several groups using enterocyte cell models have shown that vesicular iron transport for efflux can also be important [45,132,133,134]).

Ferroportin is also a critical control site for recycling iron according to need; its expression levels on the plasma membrane being controlled mainly by the small peptide hepcidin, a master regulator of the iron homeostasis already mentioned, which binds to it and causes its endocytic disposal (degradation in lysosomes) in macrophages and enterocytes [37,135]. Hepcidin expression by liver (and macrophages [136]) is enhanced by iron and inflammation, resulting in decreased intestinal iron absorption through diminished release from enterocytes, as well as retention of iron by macrophages and hepatocytes. Control of synthesis is however also important, since this provides the ferroportin for cellular iron efflux. In the most relevant cells involved in iron absorption and recycling (intestinal enterocytes, macrophages of spleen and liver, and hepatocytes), gene expression of ferroportin is promoted by hypoxia induced by anemia and blood loss (through HIF1α and HIF2α transcription factors), and by iron itself—including that from heme. The iron effect occurs in part through binding of the heavy metal transcription factor MTF1 to two metal response elements in the ferroportin promoter, and also requires Zn [37]. However, it seems that at least in the macrophages, transcription is mediated primarily by heme itself rather than by the iron released from heme, while translation and protein expression depend on the iron [137]. In hepatocytes, protein expression is regulated by iron through the IRE-IRP system, since these cells express one of two alternatively spliced forms of ferroportin mRNA that has a 5′IRE (like the mRNA of ferritin) [5,37]. This does not occur in enterocytes, which have the non-IRE form. Transcription can be inhibited by the inflammatory agent lipopolysaccharide (LPS), perhaps through changes in activation of the transcription factor NRF2. Ferroportin is thought to export Fe^2+^, since copper-dependent ferroxidases are usually required to mediate ferroportin iron efflux as well as binding of the resulting Fe^3+^ to apotransferrin in extracellular fluid (see section 2). As a consequence of these regulatory processes that control deployment of ferroportin in the plasma membrane, iron is absorbed more efficiently by the intestine, released from ferritin stores, and recycled rapidly by cells of internal organs in response to iron lack/anemia/hypoxia/blood loss, while the reverse occurs when iron is readily available, storage levels are high, or the body is in a state of inflammation or infection.

## 4. Conclusions

**Figure 1:**
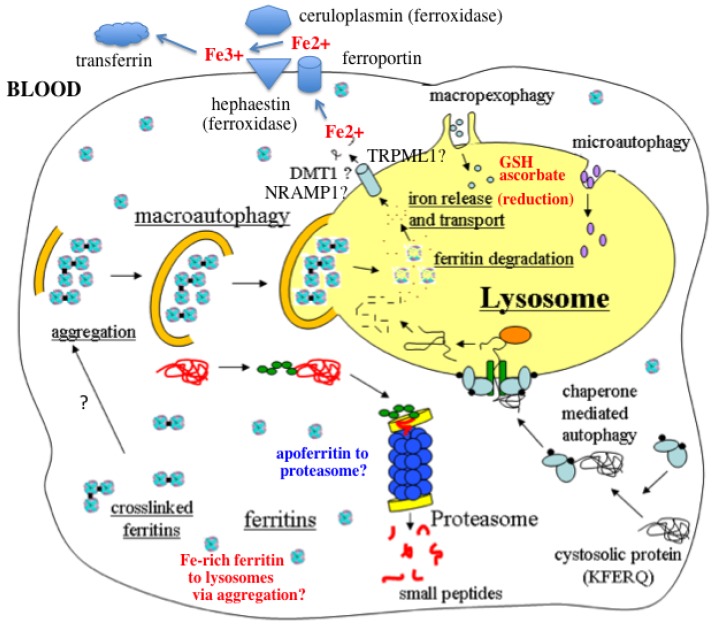
Summary of the potential and most likely steps involved in mobilization of iron stored in cytosolic ferritin when the need arises. A description of the steps is given in the text. Various forms of lysosomal autophagy including those not involved in ferritin uptake are also depicted. (Modified from E.N. Sauble [118].)

This review has considered the evidence available relating to the mechanisms by which iron stored in ferritin is mobilized by cells and organs and released for distribution to where it is needed, when called upon to do so. Figure 1 summarizes what would seem to be the most plausible steps involved that have been presented and discussed. These steps may be summarized as follows:

- In response to a signal relating to iron deprivation (resulting in a drop in the level of the labile iron pool) and/or decreased oxygen tension, cells in various organs cause ferritin in the cytosol to be taken up by lysosomes via autophagy. The form of autophagy is unclear but both macro-autophagy and perhaps also ferritin aggregation might be involved. Iron-rich ferritin may be targeted preferentially. Possibly, iron-poor and apoferritin are degraded in the cytosol by the proteasome simultaneously, to minimize reincorporation of iron into ferritin.
- In the lysosomes, the ferritin protein “shell” is degraded by cathepsins, exposing the nanocrystals of ferrihydrite inside, which dissolve in the lysosomal fluid upon reduction by glutathione, ascorbate, and other fluid constituents, the Fe^2+^ chelated by binding to them and to residual proteins present (including undegraded ferritin and perhaps also Hsp70 and metallothionein).
- Fe transporters in the lysosomal membrane, including DMT1, and depending upon the cell type also NRAMP1 and perhaps TRPML1, facilitate diffusion of Fe^2+^ into the cytosol, where it joins the labile iron pool, bound to potential chaperones that have and have not as yet been identified.
- Depending upon the cell type and perhaps also the signal, the new iron in the labile pool coming from ferritin via lysosomes is distributed to where it is needed within the cell (such as for Fe-S cluster or heme biosynthesis in the mitochondria) and/or exported into the extracellular fluid and blood via the plasma membrane transporter ferroportin and bind to the transport protein transferrin mediated by membrane-attached or soluble extracellular ferroxidases, like hephaestin and ceruloplasmin, respectively.
- Iron on transferrin then goes on mostly to develop reticulocytes in the bone marrow, but is also available to most other cells as needed, where it is taken up by receptor-mediated endocytosis of di- and monoferric-transferrin, and released from endosomes into the cytosol by DMT1 (and perhaps also other transmembrane transporters) after reduction of Fe^3+^ by Steap 3, other reductases, or endosomal ascorbate.
